# Dr. C. L. Redwine

**Published:** 1882-03-20

**Authors:** 


DR. C. L. REDWINE—WHOLESALE AND RETAIL DRUGGIST.—This gentleman has resumed the drug business in Atlanta. His experience as a Druggist, his polite and accommodating manners, his kind and affable disposition, and his great energy, will soon bring to his house a large and active trade. He is just opening up a splendid establishment at 21 Marietta street. His stock is large and varied, and the interior arrangement of his store beautiful and attractive, evincing great skill and taste in the selection and display of his goods. See his advertisement in this Journal.

